# Indents

**Published:** 1917-07

**Authors:** 


### INDENTS

☞ **Brief Articles of Technical or Scientific Value will receive notice and comment. Contributions invited.**

*Extracting Abscessed Teeth* A writer in the Dominion Dental Journal asks how is it that a patient with an ulcerated tooth will say, "Of course, you can not extract this today, as it is ulcerated. I might get blood poisoning." If there is an abscess in any other part of the body, the pus must be removed; if there is any foreign matter taken into a wound, it is removed, and why can you not have a bad tooth removed? I have extracted abscessed teeth ever since I started practice—twenty-six years ago—and have had no difficulties, if proper precautions are used. I always paint the gum with iodin, keep my instruments sterilized, and use every precaution.—*Facts.*

*A Hint About Dental Bridges* If a dental bridge is attached to a solid fixed anchorage at one end and has a loosened tooth or root for anchorage at the other, it will be a short-lived bridge. The fact that it is movable at one end makes a lever of such a bridge, and the force of mastication, being the power applied, will tear the solid fixed end loose from its anchorage, or loosen the root or break the bridge. A firm fixed anchorage at one end of a bridge will not sustain and hold firm the opposite end of the anchorage of said opposite end is loosened in the outset, or becomes loosened after the bridge has been in use. If the anchorage for one end of a bridge is not firm the anchorage for the opposite end should be correspondingly movable. If the anchorage for either end of a bridge is not firmly fixed it would be better to choose some other method for restoring or supplying the lost teeth.—H. A. Cross, *Dental Review.*

*Dental Anesthetics* 1. Before the right of the dentist to administer general anesthetics is expressly established by a judicial, legislative, or educational enactment, the only safe guide for the dentist to follow is proper qualification.

2. It would seem that qualification is not complete without the ability to render proper treatment in case of emergency arising during anesthesia or caused by anesthetics.

3. Special license, on the basis of qualification, should be created for those engaging in the administration of general anesthetics.

4. Single-handed operation is clearly illegal, a menace to life of patient, and invites trouble for practitioner.

5. As a matter of law, general anesthesia is not permissible when local anesthesia—a safer agent—can be employed for same purpose.

6. Analgesia, for excavating cavities and similar minor operations, is an unjustifiable practice.

In order not to stray from these conclusions we must leave out all selfish considerations, vague rights, blanket justifications, and consider only facts.—H. Schwamm, in *Cosmos* for May.

*Some Thoughts* Would you be satisfied:

To have instruments used in your mouth sterilized as you practice sterilization?

To have your teeth examined with the thoroughness with which you do it for others?

To have your own teeth cleaned in the way you perform this work for your patients?

With cavities in your teeth prepared and filled as you practice this part of your profession?

If it were necessary to fill the root canals of your teeth to have it done as you do it?

And abscess treatments and extractions and all of the other operations incident to your daily work, would you want them done for yourself as you do them daily?

Can you truthfully answer these questions in the affirmative?

Do you get the thought?

Are you practicing dentistry for others as you would want it done for yourself?

Are you practicing the best dentistry that can be practiced?

Are you availing yourself of the many opportunities for adding to your knowledge, such as reading of books and periodicals, attendance at society meetings and study classes?—*New Jersey Dental Journal.*

*To Make Rubber Tubing Pliable* When rubber tubing or dental rubber becomes stiff and hard to work, it may be very easily renewed or made pliable by placing it in hot water that contains about ten per cent. glycerine. This in no wise alters or damages the rubber or vulcanite.—*Pacific Dental Gazette.*

*Removing the Teeth from an Old Denture* Prepare a fifteen or twenty per cent. solution of glycerine and water, bring this to the boiling point and place the denture in it. Boil for a few minutes and the teeth may be pushed off, leaving the pins clean and the teeth uninjured.—*Pacific Dental Gazette.*

*Injecting for Conductive Anesthesia* To get results it is not necessary to inject into the nerve trunk but near it, into the connective tissue around the nerve. When this is done there is but slight danger of injuring the nerve. If injection is made into certain muscles it may be one of the causes of ill effects for a while.—J. H. Huschart, *American Dentist.*

*An Easy Way to True Carborundum Stones* Buy a common harness punch at the hardware store for ten cents. Stick the mandrel in the open end of punch, hold stones against revolving stone in lathe and you will have a true carborundum stone in a moment.—M. B. Latimer, in *Digest.*

*Use of Silver Wire*

The use of pure silver wire as a "probe" for exploring fistulous tracts may not be known to all of you. Fine wire of pure silver is used. The end is fused to a small "ball," thus softening the silver and making it flexible, at the same time completely sterilizing it.

To apply Ag. $NO^3$ to any spot within the mouth, *e. g.*, ulcerations, sensitive dentin, etc., fuse a small piece of the caustic onto the end of a silver wire, using the remaining part of the wire as a handle. One can touch the exact spot desired, without the risk of cauterizing adjacent soft tissues, as one might do when using the "stick" form of lunar cautic.—F. G. Butler-Wood, *Australian Journal of Dentristry.*

*Dental Hygienists for Maine*

The legislature of the state of Maine on April 7, 1917, enacted a law creating the dental hygienist. Section 1 reads in part as follows:

"Any registered or licensed dentist may employ women assistants who shall be known as dental hygienists. Such dental hygienists may remove lime deposits, accretions and stains from the exposed surfaces of the teeth and directly beneath the free margin of the gum, but shall not perform any other operation on the teeth or mouth, or on any diseased tissues of the mouth. They may operate in the office of any registered or licensed dentist, or in any public or private institution under the general supervision of a registered or licensed dentist."

The dental hygienist must be eighteen years of age, of good moral character, and shall have had an education equivalent to that obtained by one year's attendance in a class A high school, unless she is a graduate of a reputable training school for dental hygienists or presents a sworn statement by dentists licensed to practice dentistry in a state that she has completed a course of at least six months' training as a dental hygienist under him.

Penalties are provided for nonobservance of the law or a dentist who shall employ a dental hygienist who has not complied with all of the requirements. The state board of dental examiners examine candidates and grant a certificate if found competent, the expense of which is ten dollars. The board of dental examiners may at its discretion without examination, issue its certificate to any applicant who shall furnish satisfactory proof that she has been duly licensed to practice as a dental hygienist in another state, providing her professional education shall not be less than that required in Maine.—*Oral Hygiene.*

*Sudden Breakdown* A curious and dangerous thing about mental and physical fatigue is that a person may reach the point of complete prostration before he is aware that he is suffering from fatigue. This fact is illustrated by the sudden complete breakdown of intelligent business and professional men in the midst of a season of unusual activity. This occurrence seems to be contrary to the logical course of events; but it is easily accounted for by taking into consideration the effect of certain artificial stimuli. Intense interest, emotional or intellectual excitement, anxiety, urgent anticipation will act as effectively in the production of artificial stimulation as the drug stimulants, alcohol, caffeine, cocain, etc., and will succeed in keeping the various body functions at an apparently normal state of efficiency until the period is reached finally and suddenly where there remains practically nothing to form a basis for stimulation. At this point the functional activity ceases suddenly, and the individual is temporarily at least, a human wreck.

It is a wise man or woman who knows his or her own limitations. A nervous breakdown is not a trifle. A vacation of the right kind will often prevent it.—*Selected.*

*Some Dental Uses for Rice Flour* To make this preparation of rice flour, take impression plaster of paris four parts; rice flour one part; each to be added by measure; mix thoroughly. Place the powder in a box and label it "Crown and Bridge Impression

Plaster.'' When preparing to take impression add a little more salt to the water than you would to plaster of paris impression, as the rice flour slightly retards the crystallization of the plaster. After taking the impression, stain and pour model for crown or bridge. When model is hard, place model and impression in boiling water, and the impression will gradually disintegrate into small flakes, leaving a perfect model, crowns and teeth intact. When the impression starts to disintegrate in the boiling water, a sharp pointed instrument may be used to remove the soft parts of the impression thus hastening the separation of the impression from the model.

No injury to the model will take place in the hot water, if the model has had time to become thoroughly hard.

Rice flour is not affected by cold or luke-warm water. Hot water causes the rice granules to increase in size.—C. H. Woolgar, in *The Dental Summary*.

NOTE. We consider it a very bad practice to get a plaster model wet until it is placed in the vulcanizer.—Editor REGISTER.

*A Peculiar Case*

About October 1, a young lady twenty-one years of age, in good health, came to me for dental work. The lower left second molar had a cavity of long standing. I devitalized it and found a large pulp stone. Immediately after devitalization, the tooth became very painful to pressure. In a few days the second bicuspid, which was a perfectly sound tooth, became elongated and painful to pressure, the first molar remaining normal. After several days the bicuspid became opaque and was opened up and pulp was practically dead. The pulp was removed and treatment was sealed in with cement. Both teeth remained painful for about two weeks. Nothing that I did for them did any good whatever. I then had a good skiagraph made of left half of mandible, but it showed no bone trouble or multiple roots. I then sealed treatment in teeth and dismissed patient for several days. On February 1, I extracted the lower left third molar, which had been giving trouble for three or four years on account of its being impacted. The third molar was perfectly normal and sound, so I ground both crown and roots down to pulp and found large pulp stone in body of pulp. The impaction has been removed thirty days, but no improvement is apparent in the other two teeth.—O. M., in *Digest*.

*Size of the British Army* "A writer in the *Lancet* gives the following method of realizing what it means to increase the British army from 805,000 to 4,000,000 men: 'There are 810,697 words in the Bible, and we had about that number of soldiers at the outbreak of war. There are also 3,566,480 letters in the Bible, and we can now consider every letter as a soldier instead of every word as heretofore. Also we may consider that the first letter of every three verses is a medical man in the Royal Army Medical Corps, for there are about 10,000 medical officers now, and there are 31,173 verses in the Bible. To take a Bible and turn over its leaves and realize that every single letter is a soldier in our army produces an impressive feeling of what fighting means.' "—*British Dental Journal.*

*Platinum* Some time past the English Minister of Munitions placed an embargo on the use of platinum for other than army purposes. No one is allowed to use platinum in the manufacture of artificial teeth or jewelry. A recent order prohibits the use of lead and zinc for other than munitions, unless a special license is issued. A special concession has been granted to dental practitioners permitting them to purchase twenty-eight pounds of lead per month without making an application for a license to do so.

Having entered the conflict of nations, the time is undoubtedly coming when the use of platinum for other than war purposes will be prohibited in America. The present quotation is $6 a pennyweight, which makes it $120 per ounce, six times the value of pure gold. A set of fourteen porcelain teeth with platinum pins is quoted at $7. However, with flour at $15 a barrel, the common spud at $4 a bushel, cabbage at $120 a ton, and the humble beans of commerce costing $266 per ton, thus enabling the gentlemanly farmer to drive in with a bushel of beans and purchase a ton of coal, the dental profession has nothing to complain of.—*Oral Hygiene.*

*"Our Esteemed Contemporary"* The editor of *The International Journal of Orthodontia,* March number, has some nice things to say in regard to our editorial "Blind Readers of the Blind." "One of the strongest and most suggestive editorials that has been published in a dental journal in many years appeared in the January, 1917, issue of *Oral Hygiene.* One is not given to expect great things editorially from a journal of the *Oral Hygiene* type. But Jesus came out of the despised Nazareth, and surprises in journalism, as well as in politics, are not impossible."

This is a kiss, also some kick. Thanks for the kiss.

But, why so sad when it comes to *Oral Hygiene?* We admire *The International Journal of Orthodontia* for its clean-cut editorial matter, good printing, and paper; but will not admit that it is in any way in a superior class. Why all of this "holier than thou?" The publishers of the *International* sell books; *Oral Hygiene* sells dental instruments and appliances. Neither would be possible without its advertising pages; both are commercial. But—"Tell it not in Gath." Every dental publication dependent on its advertising pages for support is "commercial"—whatever that may mean. *Oral Hygiene* has no apologies to make; it has served the profession to the best of its ability without fear or favor, and will so continue.—*Oral Hygiene.*

*Estimating the Fee*

I can not agree with a certain "business efficiency" idea which seems to have the approval of some dentists and is being strongly urged by several really conscientious students of business in dentistry regarding the estimated fee for "fixing up the whole mouth." Advocates of the notion that the patient should be pressed into having everything in the mouth attended to certainly are working along commendable lines, but I take issue with the so-called "salesmanship" which includes a definite price for doing more than one operation, and especially in cases where many teeth are involved.

One of these ideas is being presented like this: Examine the patient's whole mouth, note everything there is to do to "fix up the whole mouth," then stand before the patient and talk health, hygiene, value of food being properly chewed, appearance, etc., etc., and when the climax of the salesmanship address is reached, name a price that is definite, by having noted the teeth to be repaired and adding ten per cent. to "what you see" so that any error in overlooking something shall be taken care of.

I maintain that the man who can examine a mouth with an explorer and mouth mirror and can properly diagnose and decide on treatments, not knowing where pulps are exposed or not, if several treatments will be needed to leave apical tissues in proper state of repose or just one, whether secondary dentin will require fifteen minutes or three hours in some crooked root, to say nothing about the uncertainties encountered in depth of decay, which sometimes must change the dentist's first intention, causing him to abandon the making of a filling for an inlay or crown; I maintain that he who can do this thing is to my mind not making an estimate at all, but is selling the patient his guesswork.

Cavities are overlooked; hopeless teeth are sometimes discovered only after some work has been done on them; many things can and do enter into the progress of dental work which alter the original outline, and while every patient should have some idea of the cost, has a right to ask it and the dentist should do his best to be reliable in his "probable cost price," the patient should be given to understand that unknown conditions are common and that any variation from what seems to be indicated will be charged for and done to the best of the operator's ability. And this last "best" is much more liable to be done when new conditions are discovered if the workman is being paid well, than to be doing something which was not covered fully by that ten per cent. in his estimate; and when he is paid for his labor he will not be dishonest with his patient if it took less work than was expected when the price was set.—S. A. Allen, in *Nebraska Dental Journal.*

*A Good Practical Hint*

In cervical cavities, or anterior ones that can be drilled from the labial side, I have the patient clench the teeth tightly on a block directly against the tooth being operated on, which gives them their natural desire to "clench their teeth" when in pain; this steadies them greatly, and has a decided influence in deadening the vibration, also the pressure at the apex reduces the pain itself very greatly.

A block in which several teeth of one jaw applied in antagonism to one in the other greatly increases this deadening and makes it possible, by holding a great pressure for a minute or so, even in occlusal cavities, to drill after pressure is removed, with little pain.—C. B. Branson, in *Digest.*

*Measures That Protect the Eye*

If you stop to think what the world would be to you if you were deprived of your eyesight, the thought may lead you to be a little more careful as to how you treat your eyes. The automobile is responsible for much trouble. A strong current of air like the rush of air in an auto, especially with the windshield down, is too much for any unprotected eye.

Goggles or glasses are needed in automobiling. The veils that women wear, even those of coarse mesh, protect the eyes from dust, but in strong currents of air, the close tissue veils or glasses are required. In going upon the water or walking on pavements that reflect the heat, wear colored glasses to protect the eyes. We may even say, to protect the brain, because severe headaches, with nausea and prostration in summer, are often due to dazzling light and reflected heat.

People who work in offices and other confined occupations often get headaches and other disorders from tired eyes. The eyes should not continually work with the same focus for hours at a stretch. Sailors, farmers, hunters and those who live much in the open rarely have eye ailments, because the focus of the eye is continually changed and so the ciliary muscles are relieved.—*The Healthy Home.*

